# Food cravings, appetite, and snack-food consumption in response to a psychomotor stimulant drug: the moderating effect of “food-addiction”

**DOI:** 10.3389/fpsyg.2014.00403

**Published:** 2014-05-08

**Authors:** Caroline Davis, Robert D. Levitan, Allan S. Kaplan, James L. Kennedy, Jacqueline C. Carter

**Affiliations:** ^1^Kinesiology and Health Science, York UniversityToronto, ON, Canada; ^2^Centre for Addiction and Mental HealthToronto, ON, Canada; ^3^Department of Psychology, Memorial University of NewfoundlandSt. John’s, NL, Canada

**Keywords:** food cravings, appetite, food consumption, psychomotor stimulant, food-addiction

## Abstract

There is mounting evidence that many highly processed foods have addictive properties, and that some cases of compulsive overeating resemble an addiction disorder. While support for the *Yale Food Addiction Scale* (YFAS) as a valid diagnostic tool has been impressive and continues to increase, to date, no research has examined the food-addiction construct in response to an actual food stimulus, and in relation to direct measures of appetite and food consumption. As part of a larger community-based study of overeating in healthy adults who were predominately overweight and obese (aged 25–50 years), 136 participants completed the YFAS, of whom 23 met the diagnostic criteria for food-addiction. They took part in a 2-day, double-blind, cross-over, single-dose drug challenge using a psychomotor stimulant (methylphenidate) and placebo. Participants were first assessed on ratings of appetite and food cravings after holding and tasting their favorite snack food, after which they were able to eat all or part of the snack, as they wished. Three separate repeated-measures analysis-of-variance procedures were carried out, each with two between-subjects factors (Diagnosis: food-addiction vs. non-food addiction) and (Sex: male vs. female) and 1 within-subjects factor (Days: drug vs. placebo). As anticipated, for all three dependent variables, there was a significant main effect for Days with a response decrease from placebo to the drug condition. With respect to *food cravings* and *appetite ratings*, results indicated that the food addiction group had significantly higher scores on both variables. For *food consumption*, there was a significant Days × Diagnosis interaction whereby the food-addiction group showed no food-intake suppression across days compared to the non-food-addiction group who demonstrated a significant decrease in snack-food consumption with methylphenidate. The finding that the food-addiction group was resistant to the food-intake suppression typically induced by a dopamine agonist supports evidence of dopamine signaling-strength differences in individuals with compulsive overeating compared to those without this disorder. This represents the first demonstration that individuals defined by their food-addiction status have a unique pattern of food-intake following a pharmacologic challenge with such agents.

## INTRODUCTION

In its recently released 5th edition, the *Diagnostic and Statistical Manual of Mental Disorders* (DSM-5) has acknowledged the existence of behavioral addictions for the first time (American Psychiatric Association, 2013). Currently, however, pathological gambling is the only one listed in the newly labeled “non-substance-related disorders” category. Although other excessive behaviors related to sex, exercise, eating, and shopping had been considered for inclusion, none was deemed to have sufficient peer-reviewed evidence for identification as a mental health problem at the time of publication (Potenza, 2014). Of these conditions, the one receiving the most discussion and research investigation in recent years is *food addiction* – the rather unsuitably named^1^ syndrome describing compulsive overeating accompanied by strong cravings and extreme difficulty in abstaining from highly palatable fare. To illustrate, a keywords search in *Web of Science* (an online scientific citation indexing service) for the year 2013 – using the terms “food addiction,” “sex addiction,” and “shopping addiction,” consecutively – rendered 48, 8, and 0 citations, in that order.

The growing legitimacy of the food-addiction concept has been heavily influenced by the premise that hyper-palatable foods, rich in sugar, fat, and salt, have the potential to foster excessive consumption and a state of dependence (Gearhardt et al., 2011a; Davis and Carter, 2014), and that some cases of compulsive overeating have striking clinical and neurophysiologic similarities to drugaddiction (Davis and Carter, 2009; Davis, 2013). Compelling preclinical research laid the groundwork and a solid foundation of evidence for biobehavioral parallels between the excessive consumption of sugar and fat and that of addictive drugs like cocaine and heroin. Readers are referred to several excellent reviews of this body of research (Avena et al., 2008, 2012; Corwin et al., 2011). The systematic study of clinical cases of food addiction came somewhat later, but has increased rapidly. This work began to flourish with the development of the *Yale Food Addiction Scale* (YFAS; Gearhardt et al., 2009) – a diagnostic tool based on the seven DSM-IV (American Psychiatric Association, 1994) symptom criteria for substance dependence, with the word “food” substituting for drugs in the questionnaire items. To date, studies have found substantial co-morbidity between binge eating disorder (BED) and YFAS food addiction, in addition to many shared psychological and biological risk factors (Davis et al., 2011; Gearhardt et al., 2011b, 2012). An even greater overlap was found in an earlier study of women diagnosed with BED where 92% of the sample met the DSM-IV criteria for dependence during a structured telephone interview – again when food replaced the drug/substance nomenclature in the assessment questions (Cassin and von Ranson, 2007). A recent qualitative study also confirmed that a high proportion of obese women with and without BED endorsed DSM symptoms of substance dependence when food was the “substance” in question (Curtis and Davis, 2014). These women felt that “loss-of-control” overeating, the inability to stop this behavior despite strong wishes to do so, and extreme cravings were the characteristics of their disorder which most resembled an addiction.

The first case-control study of food addiction in obese men and women found that those who met the YFAS diagnostic criteria had a significantly greater prevalence of BED than their age- and weight-matched counterparts (Davis et al., 2011). They also reported more intense trait-related food cravings and greater emotional and hedonic overeating than the control participants. Other research has found similar results using the YFAS symptom score (Meule et al., 2012). In addition, preliminary genetic evidence demonstrated that a composite polymorphic index of elevated dopamine signaling strength was greater in those who met the YFAS criteria for food addiction, and this profile score correlated positively with binge eating severity, food cravings, and emotional eating (Davis et al., 2013). Together these results support the view that risk for food addiction is greater in those with a hyper-sensitivity to reward and a greater appetitive motivation for reinforcing stimuli. In a study of weight-loss treatment-seeking adults, YFAS-symptom scores were also associated with lower weight loss after several weeks of treatment, suggesting that food addiction, with related signs of tolerance and withdrawal, may undermine efforts to lose weight in those trying to adopt better eating habits (Burmeister et al., 2013). A later study, however, failed to replicate these results (Lent et al., 2014).

In a recent general-population study, adults who met the YFAS criteria for food addiction had significantly higher body mass index (BMIs) and a greater percentage of adipose tissue compared to their non-food-addicted counterparts (Pedram et al., 2013). They also self-reported eating more calories from fat and protein. In addition, it was found that overweight and obese women had a significantly higher prevalence of food addiction than weight-matched men. Interestingly, this sex bias reflects the pattern of findings from drug-addiction research. For example, while drug abuse has traditionally been more widespread in men than in women (Wittchen et al., 2011), the gap seems to be narrowing, suggesting that earlier differences may simply reflect variation in opportunity and gender-biased expectations rather than in vulnerability (Becker, 2009; Colell et al., 2013). Indeed, it appears that many addiction risk factors are greater in women than in men. Women tend to increase their rate of drug consumption more quickly than men, are more likely to relapse, and to have longer periods of drug use before their next attempt at abstinence (Elman et al., 2001; Evans and Foltin, 2010) – a phenomenon known as *telescoping*, which describes an accelerated progression from the commencement of drug use to the development of dependence and admission to treatment (Greenfield et al., 2010). Women who abuse drugs also report more severe cravings and subjective drug effects than their male counterparts (Back et al., 2011), and this pattern seems to be similar for most addictive substances (Becker and Ming, 2008).

There is now compelling evidence that the cravings for addictive drugs and for hyper-palatable foods are fostered by similar biological mechanisms whereby excessive consumption of either provokes neuro-adaptations resulting in a *blunted* dopamine signal in brain reward circuitry – in particular, the nucleus accumbens and the ventral tegmental area (VTA; Volkow et al., 2013). Excessive consumption also contributes to a heightened motivational salience for the reward, which, together with dopamine down-regulation, increases the “wanting,” or an intense craving, for the substance in question (Robinson and Berridge, 2013). *Cravings* are therefore an important component of the addiction process, especially because they appear to increase the risk for relapse following abstinence (Sinha et al., 2006). In this context, it is noteworthy that conventional weight-loss programs, including dietary restriction and increased physical activity, are typically ineffective in the long term for patients with problematic overeating and obesity (Begin et al., 2006; Mann et al., 2007). Indeed, numerous obesity studies have linked cravings to overeating and weight gain, to the lack of success in attempts to restrict calories, and to early drop-out from bariatric treatment programs (Batra et al., 2013).

Not surprisingly, given the down-regulatory neurophysiologic processes in addiction, treatments that serve to increase dopamine signaling have shown some success in reducing episodes of overeating. For example, in a randomized-control trial, pharmacotherapy with an amphetamine-based stimulant medication was effective in lowering the frequency of binge episodes in those with compulsive overeating (Shaffer, 2012; Gasior et al., 2013). Similar medications have also been successful in producing weight loss in those with intractable obesity and co-morbid symptoms of attention deficit/hyperactivity disorder (ADHD; Levy et al., 2009). Likewise, laboratory studies of single-dose administrations of methylphenidate [a dopamine transporter (DAT) blocker] have also shown decreased food cravings and food consumption in obese adults and those with BED (Leddy et al., 2004; Goldfield et al., 2007; Davis et al., 2012). And finally, non-invasive dorsolateral prefrontal cortical (DLPFC) neurostimulation – a procedure which is believed to increase dopamine excretion via interconnections between the DLPFC and the VTA and nucleus accumbens – has also produced reductions in drug and food cravings (Jansen et al., 2013).

### THE PRESENT STUDY

Although various studies have used food-related cues in their experimental paradigms (Gearhardt et al., 2011b; Meule et al., 2012), to the best of our knowledge, there are no *objective* food consumption studies in human food-addiction research. Since self-report measures of food intake may be subject to biased recall, it is also important to have objective food-intake data for a more complete understanding of the phenomenology of disordered (and other) eating behaviors. The purpose of the current study was therefore to compare appetite, cravings, and consumption between adults diagnosed with and without YFAS food addiction, in response to a snack-food challenge following a single-dose administration of methylphenidate versus placebo. Given the generally experienced, appetite-suppression, effects of stimulant drugs, and their suggested therapeutic use in reducing binge episodes (Levy et al., 2009; Shaffer, 2012; Gasior et al., 2013), the primary purpose for including the drug challenge in the study protocol was to identify possible factors moderating response magnitude to methylphenidate, given the considerable response variability among patients taking such medications^2^.

Sex differences were also assessed in this 3-way mixed model, double-blind, cross-over design. It was anticipated that the food-addiction group would report greater appetite and food cravings and consume more of their favorite snack during the placebo condition than the non-food-addiction group. Another goal of this study was to investigate whether food addiction moderated the appetite-suppression effects typically found following administration of methylphenidate. It was speculated that the stronger appetitive responses to food associated with food-addiction (Davis et al., 2013) might buffer the normally experienced suppression effect from methylphenidate. Finally, and based on other sex differences in clinical and pre-clinical drug-response research, it was predicted that females would be more responsive to the appetite and food consumption suppression effects of methylphenidate than males.

## MATERIALS AND METHODS

### PARTICIPANTS

As part of a larger community-based study of overeating in healthy adults who were predominately overweight and obese and between the ages of 25 and 50 years, 136 participants (women = 92; male = 44) completed the YFAS, of whom 23 met the diagnostic criteria for food addiction. The food-addiction group had a mean BMI of 34.6 ± 7.0 and a mean age of 33.9 ± 5.9 years compared to the non-food-addiction group with a mean BMI of 33.8 ± 8.4 and a mean age of 32.4 ± 6.6 years. These values were not significantly different. Participants were recruited from posters, newspaper advertisements, and online sites like Craigslist and Kijiji. Inclusion criteria were residence in North America for at least 5 years and fluency in written and spoken English. Women were also required to be pre-menopausal as indicated by the reporting of regular menstrual-cycles. Exclusion criteria were a current diagnosis (or history) of any psychotic disorder, panic disorder, or substance abuse as diagnosed by the Structured Clinical Interview for DSM-IV (SCID), any serious medical condition like cancer, or heart disease, and any medications contraindicated for methylphenidate (e.g., certain antidepressants like Wellbutrin). Twenty-six percent of the food-addiction group, and 20 percent of the control group were regular smokers. Women who were pregnant or breast-feeding, or who had given birth within the past 6 months were also excluded. This study was approved by the institutional Research Ethics Boards and was carried out in accordance with the Declaration of Helsinki.

### MEASURES

#### Food addiction

Food addiction was diagnosed by the 25-item YFAS (Gearhardt et al., 2009) – a self-report questionnaire measure – using the dichotomous scoring procedure proposed by its authors. Based on the DSM-IV (American Psychiatric Association, 1994) criteria for substance dependence, a diagnosis is given if the respondent endorses three or more of the symptom subscales “over the past year” and if s/he also confirms the “clinically significant impairment” criterion.

#### Food cravings

Food cravings were assessed by the 15-item *State* version of the *General Food Cravings Questionnaire* (Cepeda-Benito et al., 2000). This well-validated scale (Nijs et al., 2007) was personalized for each participant by replacing the general words “tasty food” with the specific snack-food each participant had identified. For example, where appropriate, item one was changed from “I’m craving tasty food” to “I’m craving potato chips,” and so on. The alpha coefficients for Day 1 and Day 2 were 0.93 and 0.92, respectively.

#### Appetite ratings

Appetite ratings were assessed, after participants had been given their snack, by the sum of 3 Likert-scale questions, each scored from 1 (“not at all”) to 10 (“a great deal”): (1) How hungry does it make you feel to see your favorite snack? (2) How much would you like to eat some of your favorite snack – even just a small portion? (3) Now that you’ve had a taste of your favorite snack, how strong is your desire to have some more? After the second question, participants were asked to take a few bites of their snack, before the third question was asked.

#### Snack-food consumption

Snack-food consumption was quantified as the weight of the snack (to the nearest gram) at the end of the session subtracted from the initial weight of the snack. The amount consumed was then converted to a percentage of the initial snack weight. For example, a score of zero indicated that none of the snack was eaten and a score of 100 indicated the entire snack was eaten.

### PROCEDURES

The data reported in this study are part of a larger and more extensive protocol involving three separate assessment sessions. They comprise a sub-set of participants who were assessed on the YFAS. Using a randomized, double-blind, cross-over design, participants were administered either a dose of oral methylphenidate equivalent to 0.5 mg/kg body weight (to a maximum dose of 55 mg), or placebo, at the same time of day and the same day of the week, separated by 1 week. This dose was selected because it has been used successfully in other drug challenges with healthy adults (Volkow et al., 2001). Methylphenidate was titrated for BMI because of evidence-based recommendations that this compound should be prescribed on a weight-adjusted basis (Shader et al., 1999). Methylphenidate and placebo were packaged in identical colored capsules to prevent detection of the drug by taste or color.

#### Day 1

Demographic information was obtained, a psychiatric assessment was administered, and questionnaire measures were distributed to be completed at home and returned at the second assessment. Participants had height and weight measured, blood pressure was taken, and an electrocardiogram was carried out to confirm eligibility for the subsequent drug challenge sessions. Participants were also asked to indicate their “favorite snack food” in preparation for the food challenge taking place at the 2nd and 3rd session. The most commonly chosen snacks were potato chips, chocolate bars, and cookies. For a more detailed explanation of the protocol see Davis et al. (2012).

#### Days 2 and 3

Both 2.5-h sessions were scheduled at the same time of day and the same day of the week, separated by 1 week. Prior to each session, participants were told to eat a normal meal 2 h before their appointment and to abstain from drinking any caffeinated beverage or smoking nicotine on the day of, and prior to, their appointments. These dietary restrictions were confirmed on each testing day. Upon arrival at the laboratory, a 10-item, visual-analog, mood adjective scale was given at baseline and every 15 min after the ingestion of the capsule. The peak uptake for methylphenidate is approximately 1 h. During that time, participants were seated in a quiet area and encouraged to occupy themselves with reading materials. About an hour and 15 min after the ingestion of the capsule, participants were given their favorite snack-food to hold, and the appetite rating questions were asked, after which they were given the craving questionnaire to complete. Participants were then told that the study tasks were finished and they could eat as much of their snack as they wished. At this point more than 3 h had elapsed since their last meal.

## RESULTS

In order to assess whether there were group differences in the initial weight of the snack food – since each participant chose his/her own – a 2 (Sex) × 2 (Diagnostic Group) analysis of variance (ANOVA) was carried out. Results confirmed there were no differences between men and women (*p* = 0.828) or between the food-addiction and the non-food-addiction groups (*p* = 0.413), and there was no significant interaction between these two variables (*p* = 0.974).

### REPEATED MEASURES ANOVA

Three separate 2 × 2 × 2 mixed model, repeated measures ANOVAs were computed – one for each of the dependent variables: food cravings, appetite ratings, and percentage of food consumed. There was one within-subjects factor (Days: placebo vs. drug) and two between-subjects factors: (Sex: male vs. female) and (Diagnostic Group: food-addiction vs. non-food-addiction)^3^.

**FIGURE 1:**
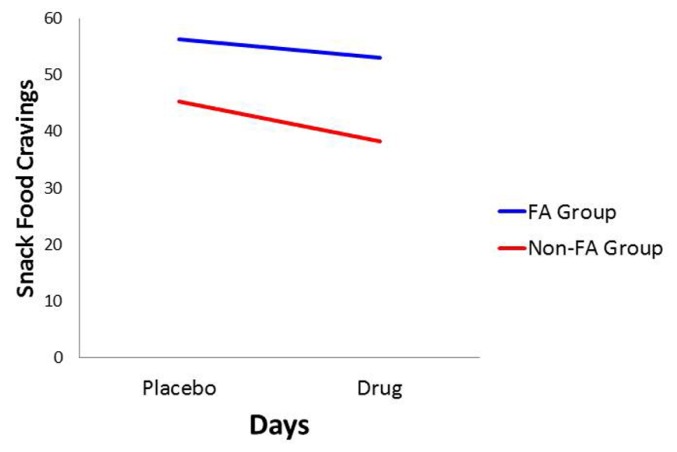
**Plot for the Diagnostic Group × Days interaction with food cravings as the dependent variable**.

**FIGURE 2:**
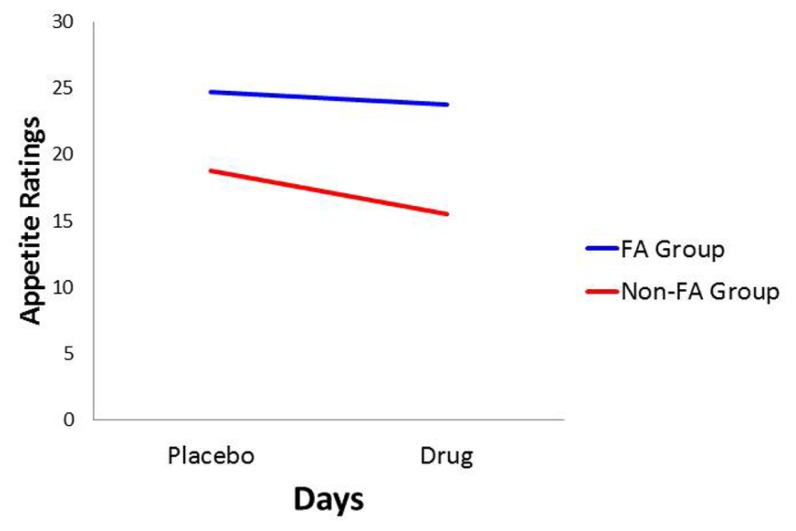
**Plot for the Diagnostic Group × Days interaction with appetite ratings as the dependent variable**.

With *food cravings* and *appetite ratings* as the dependent variables, there was a significant main effect for Diagnostic Group (*p* < 0.0001 for both: $\eta_{p}^{2}$ = 0.157 and 0.128, respectively) with the food-addiction group reporting higher scores than the non-food-addiction group. In both cases, there was also a significant main effect for Days, indicating a diminution in scores in the drug condition compared to the placebo condition (*p* = 0.006 and 0.031, and $\eta_{p}^{2}$ = 0.056 and 0.035, respectively), but these decreases on the drug day were not significantly different between those with and without food addiction. These results are presented graphically in **Figures 1 and 2**.

In conformance with statistical convention, the absence of a significant interaction between the food-addiction and non-food-addiction variable and the placebo vs. drug variable precludes the legitimate testing of *post hoc* individual-Group comparisons across Days. It is important to note, however, that this interaction is testing the significance of the *difference in slopes* between the two groups. It is not testing whether either slope is different from zero. In this case, a slope not different from zero indicates no drug-suppression effect. Since the primary question of interest in the current study was whether one or both of the food-addiction groups displayed a suppression effect – not simply whether they differed from each other – a test of simple slopes was carried out for each group, strictly acknowledging that the results are investigational and preliminary. In the non-food-addiction group, the decrease from the placebo to the methylphenidate condition for appetite ratings and food cravings was statistically significant in both instances (*p* < 0.0001: $\eta_{p}^{2}$ = 0.260 and 0.1.86, respectively). In the food-addiction group, neither comparison was statistically significant (*p* = 0.257 and 0.198, respectively).

There were no significant differences between men and women, nor did they differ on their food cravings and appetite ratings when they were taking the placebo or the drug.

**FIGURE 3:**
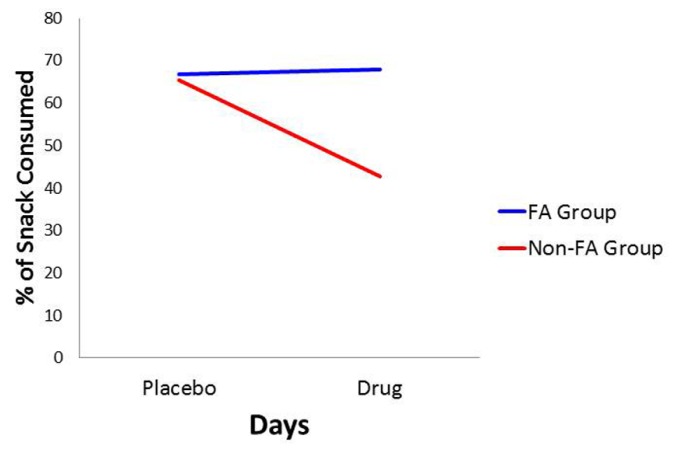
**Plot for the Diagnostic Group × Days interaction with percentage of snack-food consumption as the dependent variable**.

**FIGURE 4:**
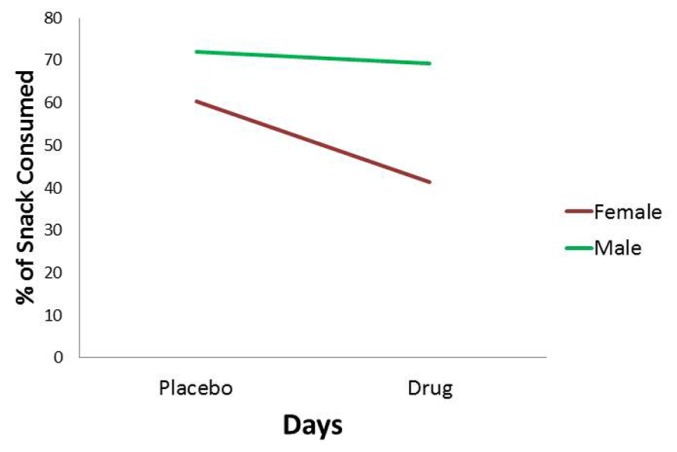
**Plot for the Sex main effect with percentage of snack-food consumption as the dependent variable**.

For the *percentage of consumed snack-food*, there was a statistically significant interaction between Diagnostic Group and Days (see **Table 1**). As indicated in **Figure 3**, and according to *post hoc* comparisons, the food-addiction group showed no reduction in food-intake in the drug condition from the placebo condition, while there was a significant decrease in the non-food-addiction group (*p* < 0.0001: $\eta_{p}^{2}$ = 0.276). There was also a significant main effect for Sex (*p* = 0.022: $\eta_{p}^{2}$ = 0.039) with men consuming a greater percentage of their snack than women (see **Figure 4**)^4^.

**Table 1:** Summary statistics for the within subject contrasts for the 2 [Days] × 2 [Sex] × 2 [Diagnostic Group] ANOVA with Food Consumption as the dependent variable.

Source	Type III SS	df	*f*	*p* =
Days	1464.50	1	5.25	0.023
Days × Sex	833.22	1	2.99	0.086
Days × Diagnosis	1611.53	1	5.78	0.018
Days × Sex × Diagnosis	704.36	1	2.53	0.114
Error (Days)	36512.17	131	–	–

### MOOD RATINGS

In light of the food consumption group differences in response to methylphenidate, it was decided to assess whether this finding reflected variation in subjective mood response to the drug, possibly as a result of differences in uptake or metabolism. The first item on the visual-analog scale, which was given every 15 min after ingestion of the capsule, asked participants whether they felt any mood or emotional changes that could be attributed to taking a stimulant medication. Participants indicated their response by making a pencil mark on a line 147 mm long, where the left end of the line indicated “no effect” and the right end of the line meant a “very strong” effect. Scores therefore varied between 0 and 147.

**FIGURE 5:**
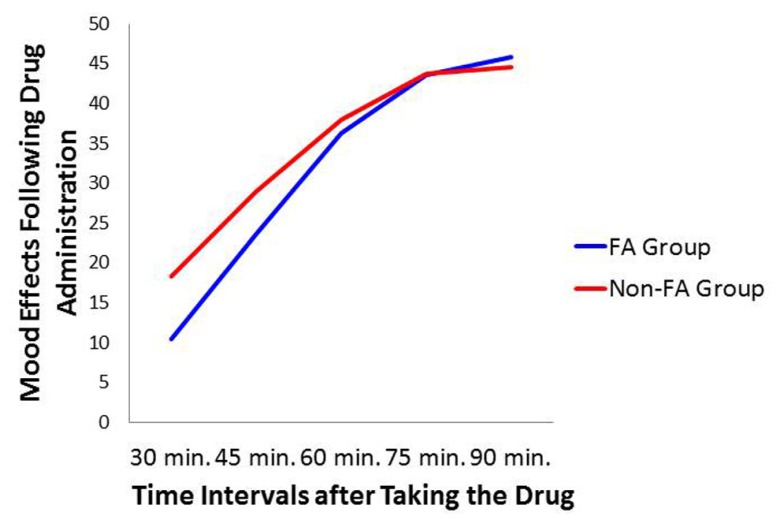
**Plot for the Diagnostic Group × Time Interval relationship with mood ratings on the drug day as the dependent variable**.

Repeated measure ANOVA was employed to assess the rating at time periods: 30, 45, 60, 75, and 90 min after ingestion of the capsule on the drug day. Similar to the previous analyses, between-subjects factors were Sex and Diagnostic Group. Results indicated a significant effect across time periods (*p* < 0.0001: $\eta_{p}^{2}$ = 0.254) with a linear increase which stabilized at 75 min post ingestion. There were, however, no differences between the food-addiction and the control groups, nor a difference between men and women. There was also no interaction between these two variables. **Figure 5** depicts the Time effect with separate lines for the food-addiction and the non-food-addiction groups. It is noteworthy that the peak subjective effect of the drug occurred at approximately 75 min after the capsule was taken – the time the snack-food challenge took place – after which the effect appeared to plateau in both groups.

## DISCUSSION

This study represents the first empirical support for the food-addiction theory, based on *actual* food intake. Results demonstrated significant eating-related differences in response to a snack-food challenge between those diagnosed with YFAS food addiction and the non-diagnosed control group. The former reported stronger food cravings and greater appetite ratings following a taste of their favorite snack, and these differences remained stable in both the placebo and the methylphenidate conditions. While there was an overall diminution in these self-reports from placebo to drug, as was expected, this effect was driven primarily by a decrease in the non-food-addiction group, since there was no diminution among those with food addiction. With respect to food consumption, there was a significant interaction between Diagnostic Group and Days, again showing a substantial decrease in snack-food consumption in the non-food-addiction group, while there was no change in the food-addiction group.

Interestingly, and contrary to prediction, there was no difference between the food-addiction and the non-food-addiction groups in the percentage of food consumed in the placebo condition. Since appetite ratings and food cravings were both higher in the food-addiction group after the snack food was presented, it is difficult to explain why their food intake was also not greater on the drug-free testing day. One possibility is that a ceiling effect accounted for the null finding. Specifically, each participant was given a *single* snack item such as a chocolate bar, a cookie, or a small bag of chips. When analyzing the data, it was noted that a large proportion of the sample consumed the entire snack in the placebo condition – viz. 55% of the food-addiction group and 44% of the controls, compared to 45 and 25% respectively in the drug condition. If the size of the snack had been larger, thereby providing an opportunity for greater variability at the high-consumption end of the distribution, it is possible that placebo group differences may have emerged.

To summarize, in response to the methylphenidate challenge, the food-addiction group appeared resistant to the typical appetite-suppression effects of this drug. One can only speculate on the mechanisms underlying these results. Methylphenidate is lipophilic and therefore some of the drug may be sequestered in fat tissue. However, since the mean BMI values were equivalent in the two groups, differences in fat mass are unlikely to account for the observed group effects. In addition, the absence of any difference between the groups in the reporting of subjective drug effects, or on the timing of the peak subjective effects (see **Figure 5**), suggests that metabolic variation is unlikely to account for the appetite/eating group differences. Because methylphenidate’s mechanism of action is very similar to that of cocaine – both block the DAT – some biological insights may be gleaned from preclinical research using a strain of cocaine-insensitive mice. The DAT-CI is a knock-in mouse line containing three point mutations in the DAT gene. This genetic alteration reduces DAT function and thereby leads to a hyper-dopaminergic state as reflected by heightened spontaneous locomotion in these animals compared to wild-type strains (O’Neill and Gu, 2013). Since inhibition of the DAT is necessary for a response to cocaine, as expected these genetically modified animals also do not display an increase in locomotion following cocaine administration, nor a conditioned place preference (O’Neill et al., 2013).

It is relevant that in previous human research we found evidence of an increased striatal dopamine signal – as indexed by a multi-locus genetic profile – in a group of adults diagnosed with YFAS food addiction compared to their age- and weight-matched counterparts (Davis et al., 2013). These findings are consistent with behavioral evidence that hyper-responsive brain reward mechanisms may serve as a risk factor for the tendency to over-consume highly palatable foods. Like the DAT-CI mice, individuals with a predisposition to elevated dopamine activity may also be relatively inured to the typical effects of stimulant drugs like cocaine and methylphenidate. Our results may therefore have potential clinical implications because methylphenidate is the first-line drug treatment for adults with ADHD, and similar stimulant drugs have recently shown some efficacy in reducing binge episodes in adults with BED (Shaffer, 2012; Gasior et al., 2013). Moreover, in light of the evidence that food addiction may reflect a more severe form of BED (Davis, 2013), the results of this study could assist in the development of personalized treatment management for patients with compulsive overeating. Indeed, many patients who use stimulant drugs therapeutically are non-responsive or discontinue treatment because of negative side effects – findings which suggest that pharmacogenetic research is needed to better understand the factors that influence drug effectiveness and toxicity. Regrettably, few adult studies have been conducted in this field, although some positive findings have identified influential markers on the DAT1 gene in relation to drug responsiveness (Contini et al., 2013).

With respect to sex differences, we found little support for our prediction that females would be more responsive to methylphenidate than males. Considering there were no Sex × Days interactions, our results do not mesh well with pre-clinical research demonstrating a stronger response to methylphenidate in females compared to males. For example, adolescent female rats showed a more robust sensitization to a dose of methylphenidate compared to their male counterparts (Brown et al., 2012), although later research found no sex differences in conditioned-place preference using the same drug (Cummins et al., 2013). It is also noteworthy that these drug effects were moderated by the strain of rats and by the drug dose (Chelaru et al., 2012).

Overall, the present study has added to the growing body of research supporting the validity of the food-addiction construct. To the best of our knowledge, this is the first study to use a well-controlled, laboratory-based, food challenge to make eating-related comparisons between adults with and without YFAS-diagnosed food addiction. In accord with our previous evidence of strong links between food addiction and trait-like food cravings (Davis et al., 2011), the current study also found elevated state-related food cravings in response to the physical presence of a highly palatable snack, which participants were asked to taste and invited to eat. Nevertheless, it is important to emphasize that replication is needed with larger samples of individuals meeting YFAS criteria for food addiction in order to improve confidence in the outcomes of this research. In the present study, the sample lacked adequate power to test the Sex × Diagnostic Group interaction due to small frequencies in some of the cells. Future researchers are also encouraged to provide a greater quantity in the snack-food challenge in order to increase the range of food-consumption scores. In addition, larger samples will allow researchers to take account of menstrual-cycle status in female participants since estrogen and progesterone levels are known to influence response to stimulant drugs (Evans and Foltin, 2010). And finally, we encourage studies going forward to search for mechanisms to explain the apparent food-related insensitivity to methylphenidate in those with YFAS food addiction by using sophisticated brain imaging techniques.

## Conflict of Interest Statement

The authors declare that the research was conducted in the absence of any commercial or financial relationships that could be construed as a potential conflict of interest.

## Footnotes

1 There has been justified criticism of the contiguousness of the words “food” and “addiction” in this putative diagnostic label because the word “food” connotes substances essential for survival and fundamental to human existence, while “addiction” implies psychopathology and even antisocial behavior. More appropriate perhaps would be terms like “hyper-palatable processed food” or “high fat, sweet, and salty food” because those that are intensely craved and over-consumed, and that comprise most binge episodes are not grown or raised in nature. Instead they are highly processed foods, calorically dense in fat, sugar, and salt, and are almost universally perceived as very appetizing (Curtis and Davis, 2014).

2 These potential moderators included genetic factors, results for which will be published elsewhere for the larger study.

3 Each of the three repeated measures ANOVAs was re-run with BMI included as a co-variate. In each case, BMI did not correlate with the dependent variable nor were the Days × BMI interaction terms statistically significant, indicating that BMI did not contribute to the variance in the appetite, cravings, and food-consumptions variables. Therefore it was removed from the models. The values reported in the Table and the Figures are the results without BMI.

4 As a *post hoc* analysis, we investigated whether the methylphenidate effect on food-intake was associated with its effect on food cravings and appetite ratings. We calculated a difference score (placebo – drug) for each of the three food-related variables and examined their bivariate inter-correlations. The food consumption difference score was moderately correlated with the cravings and appetite difference scores (*r* = 0.39 *p* < 0.0001, and *r* = 0.35 *p* < 0.0001, respectively), which were themselves highly correlated (*r* = 0.76, *p* < 0.0001).
